# The Prevalence and Control of Lungworms of Pastoral Ruminants in Iran

**DOI:** 10.3390/pathogens11121392

**Published:** 2022-11-22

**Authors:** Salman Zafari, Sina Mohtasebi, Alireza Sazmand, Aliasghar Bahari, Neil D. Sargison, Guilherme G. Verocai

**Affiliations:** 1 Department of Medical Parasitology and Mycology, School of Medicine, Hamadan University of Medical Sciences, Hamadan 6517838736, Iran; 2 Department of Medical Parasitology and Mycology, School of Public Health, Tehran University of Medical Sciences, Tehran 1417613151, Iran; 3 Department of Pathobiology, Faculty of Veterinary Science, Bu-Ali Sina University, Hamedan 6517658978, Iran; 4 Department of Clinical Sciences, Faculty of Veterinary Science, Bu-Ali Sina University, Hamedan 6517658978, Iran; 5 Royal (Dick) School of Veterinary Studies, University of Edinburgh, Easter Bush Veterinary Centre, Roslin, Midlothian, Scotland EH25 9RG, UK; 6 Department of Veterinary Pathobiology, School of Veterinary Medicine and Biomedical Sciences, Texas A&M University, College Station, TX 77843, USA

**Keywords:** lungworms, nematoda, verminous pneumonia, protostrongylidae, metastrongyloidea, ruminants, anthelminthics

## Abstract

Lungworms of the genera *Dictyocaulus*, *Muellerius*, *Protostrongylus*, and* Cystocaulus *are common helminths of domestic and wild ruminants with substantial veterinary and economic importance. Several studies have assessed the presence and prevalence of lungworm infections in ruminants in Iran. This report compiles the available scientific information about the occurrence of lungworms in domestic and wild ruminants in Iran between 1931 and June 2022 to give an insight into their epidemiology, and where possible to describe drug treatment efficacy. For this purpose, national and international scientific databases were searched. Overall, 54 publications comprising 33 articles in peer-reviewed journals, 8 conference papers, and 13 dissertations were evaluated regarding prevalence data; and an additional 4 peer-reviewed articles were evaluated regarding drug efficacy. Seven species of lungworms, namely *Dictyocaulus filaria*, *Dictyocaulus viviparus*, *Dictyocaulus eckerti*, *Protostrongylus rufescens*, *Protostrongylus raillietti*, *Muellerius capillaris*, and *Cystocaulus ocreatus *have been recorded from different ruminant hosts in Iran. Thirty-three studies conducted on small ruminant (sheep and goat) lungworms reported prevalences of lungworm infection of 11.6%, 45.81% and 66.29% using abattoir meat inspection, Baermann technique and fecal flotation, respectively. Eight studies conducted on large ruminants (cattle and water buffalo) reported prevalences of infection of 14.83%, 13.98% and 5% using abattoir meat inspection, the Baermann technique and fecal flotation, respectively. The prevalence of infection in wild ruminants was variable across examined species; 38% in urial, 37% in wild goats, 5% in goitered gazelles and 67% in red deer, in addition to a single case report in roe deer. There are few contemporary studies assessing the efficacy of currently available broad-spectrum anthelmintic compounds against lungworms in Iran. The high prevalence of multiple lungworm species in Iran, combined with a lack of information about drug efficacy, supports the need to improve the understanding of these important nematode parasites and inform the development of sustainable control strategies. The aim of this review and meta-analysis is to provide a baseline for future conventional parasitology and next generation molecular epidemiological studies of lungworm infection in pastoral ruminants in Iran.

## 1. Introduction

Approximately, half of all creatures on earth are parasites [1]. These organisms play important roles in the regulation of host populations, food webs, and interspecific interactions [2]. In addition, they impact the health and welfare of their hosts. Domestic ruminants are widely kept for high quality protein production, but milk and meat production can be reduced as a result of parasitic infections [3,4]. Among these parasites, lungworms can cause significant economic losses, underpinning the importance of correct diagnosis, treatment, and control [5]. Lungworm species within the superfamilies Trichostrongyloidea and Metastrongyloidea are of veterinary importance in domestic ruminants in Iran. Among the Trichostrongyloidea, *Dictyocaulus viviparus* is of major importance in bovines and *Dictyocaulus filaria* in small ruminants*. Dictyocaulus* species lungworms have direct life cycles in which the first-stage larvae (L_1_) are shed in faces where they develop into infective third-stage larvae (L_3_) in the environment. L_3_ are ingested by ruminants while grazing and migrate to the respiratory system, where they mature into dioecious adults within the lumen of bronchi and bronchioles. Females shed eggs, which embryonated in the lungs, and hatch within the gastrointestinal tract, before being shed in feces. Diagnosis can be supported by larvoscopy to identify L_1_ in feces most often using the Baermann technique [6].

Among the Metastrongyloidea, *Muellerius capillaris*, *Protostrongylus rufescens*, and *Cystocaulus ocreatus* are important in sheep and goats. These lungworms have indirect life cycles, requiring gastropod intermediate hosts for the development of L_1_ to the infective L_3_ stage [6,7]. Adult females lay eggs in the terminal bronchioles and alveoli. L_1_ hatch within the airways, are coughed and swallowed together with respiratory secretions and passed, before invading susceptible terrestrial snail hosts. Development to the infective L_3_ takes a few weeks to several months, depending on lungworm species, weather conditions and the suitability of the gastropod host species. Small ruminants become infected by inadvertently ingesting snails harboring infective L_3_ [8]. Additionally, some protostrongylid L_3_ may actively escape the snails, hence small ruminants can become infected while ingesting free L_3_ while grazing [9]. Ingested L_3_ migrate to the respiratory system, maturing to dioecious adults following a prepatent period of 1–3 months. In the absence of effective treatment, patent infections may persist for several years.

Currently, the genus *Dictyocaulus* contains eight valid species: *D. viviparus* (Bloch, 1782) in large ruminants (cattle and buffaloes), *D. filaria* (Rudolphi, 1809) in small ruminants (sheep and goats), *Dictyocaulus arnfieldi* (Cobbold, 1884) in equids (horses and donkeys, *Dictycaulus eckerti* (Skrjabin 1941) in cervids, *Dictycaulus cameli* (Boev, 1951) in camels, *Dictycaulus africanus* (Gibbons & Khalil, 1988) in African artiodactylids (camels and boars), *Dictycaulus capreolus* (Gibbons & Höglund, 2002) in roe deers and *Dictycaulus cervi* (Pyziel et al. 2017) in red deers. *Dictyocaulus viviparus* infecting cattle and water buffaloes and *D. filaria* infecting small ruminants are among the most common lungworms around the world, causing economic loss and compromising animal welfare [6,10]. Host responses to infection cause bronchitis and eosinophilic pneumonia, which along with blockage of respiratory bronchioles, can result in dyspnea, coughing, pyrexia, loss of appetite, poor growth rates, and death [4,11,12]. Harsh respiratory sounds are heard on auscultation of the chest. Secondary bacterial infections may add to the pathology arising from infection [5]. Although *D. filaria* and *D. viviparus* usually infect ruminants, they have also been reported in camels [13], putatively due to cross-infection from ruminants [14]. *Dictyocaulus eckerti* (Skrjabin, 1931) is often found in small numbers in adult cervids, namely reindeer, fallow deer, red deer, roe deer, and moose, with no apparent clinical disease [15]. However, farmed deer, especially young animals in their first year of life, are highly susceptible to lungworm infection resulting in loss of productivity and fatality [15]. Cross-species transmission of *Dictyocaulus* spp. has been demonstrated between wild cervids and bovids [16].

The *Protostrongylus* genus of metastrongyloid lungworms contains at least thirteen species which *P. rufescens* (Leuckart, 1865) is the most common species infecting small domestic ruminants worldwide [17]. Although protostrongylosis is widespread, it is not considered highly pathogenic, albeit infected animals may show signs of mucopurulent nasal discharge, dyspnoea, anorexia and weight loss [18]. Nonetheless, pathological changes characterized by chronic eosinophilic granulomatous pneumonia (visible by histopathology) are frequently detected on postmortem examination [19] and are an important cause of offal rejection at meat inspection in many countries.

*Muellerius capillaris* (Müller, 1889) is present worldwide in small ruminants [20]. The presence of infection is high in goats compared to sheep [21]. Heavy infections can result in interstitial pneumonia, broncho-pneumonia, or fibrinous pleuritis [6], resulting in reduced weight gain, poor reproductive performance, depressed milk production, and increased mortality [22].

*Cystocaulus ocreatus* (Railliet & Henry, 1907) is present in small ruminants in parts of Asia, Europe, and Africa [17]. Economic losses have been reported due to both adult and developing stages in the alveoli, alveolar ducts, and bronchi [23].

By comparison with gastrointestinal nematodes, lungworms have received little attention from the scientific community of Iran. This review aims to evaluate and unfold the status of lungworm infection in domestic and wild ruminants in Iran.

## 2. Materials and Methods

International online databases prior to July 2022 (Google Scholar, Pubmed and CAB Direct) and the Iranian national online databases (Irandoc and Civilica) were searched singularly and in combination, using English, Persian and French language keywords: lungworm(s), mammals, domestic, wild, wildlife, ruminant(s), treatment; plus common and scientific names of the hosts and lungworms.

The validity, accuracy, clarity, transparency in the originality, objectivity, sampling method and diagnostic method reported in each document was evaluated by two nematode parasitologists. Validated reports were then sorted for the study area, type of host animal, species diversity and species prevalence, year of study, the number of animals studied and methods used in generating the report.

## 3. Results

**Figure 1:**
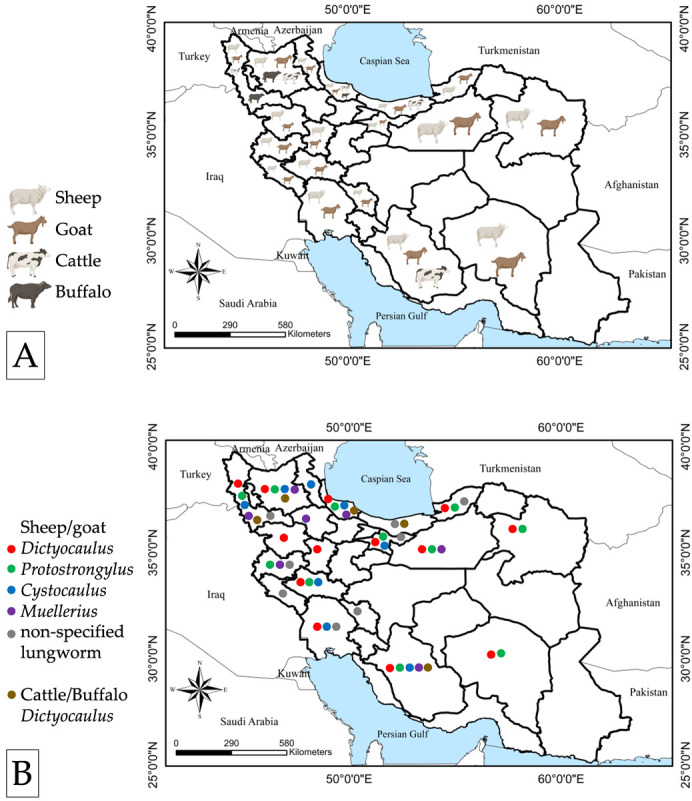
Map of Iran showing (**A**) the distribution of reports of lungworms of sheep, goats, cattle and buffaloes, (**B**) the distribution of reported lungworms of sheep, goats, cattle and buffaloes.

Since the first description of lungworm infection in Iran in 1931, a total of 60 reports were found, of which six sources were excluded in the evaluation of validity and accuracy. The 54 reports included in this review comprise 33 peer-reviewed articles [15 in English, 13 in Persian with English abstract, 5 in Persian], 8 conference papers [4 in English, 4 in Persian], 11 D.V.M. theses by veterinary students [8 in Persian, 2 in Persian with English abstract, 1 in French], and 2 Master’s theses [in Persian with English abstract]. In addition, four peer-reviewed articles [4 in English] were used to assess the efficacy of anthelmintic drugs against lungworms in large and small ruminants. No record of infection of ruminants was found in 13 provinces, in 12 of which, veterinary faculties are not present, i.e., the University of Zabol in Sistan-va-Baloochestan province has a veterinary faculty (Figure 1A).

Overall, seven species of lungworms belonging to four genera have been reported in Iran. These are *D. filaria*, *D. viviparus*, *D. eckerti*, *P. rufescens*, *P. raillietti*, *M. capillaris*, and *C. ocreatus*, *reported in* domestic sheep, goats, cattle, buffaloes and wild ruminants, namely urial (*Ovis orientalis*), wild goats *(Capra aegagrus)*, goitered gazelle *(Gazella subgutturosa)*, roe deer *(Capreolus capreolus)*, and red deer (*Cervus elaphus*) (Figure 1B).

### 3.1. Sheep and Goat Lungworms

Thirty-three publications reported lungworm infection in sheep and goats in Iran, including 21 peer-reviewed articles, 12 dissertations (11 D.V.M., 1 Master’s), and 6 conference papers. These reports depicted infection in different regions of Iran, including 17 of the 31 provinces. The first record of lungworms is the 1931 report by a French veterinarian who wrote that verminous bronchopneumonia is prevalent in sheep in Iran (Carpentier, 1931). Since then, in total, 28,964 animals were examined for the presence of infection. The majority of animals (80.6%, 23,349) had their respiratory system examined at necropsy, and 19.4% of cases (5615) were diagnosed by microscopy-based fecal diagnostic tests on apparently healthy animals. Prevalence rates of lungworm infection were 11.6%, 45.8% and 66.3% using abattoir meat inspection, Baermann technique, and fecal flotation, respectively (Table 1). One drawback of Iranian reports on small ruminant lungworms is that the plucks of sheep and goats were not inspected separately in abattoirs, because sheep and goats are slaughtered concurrently on the same line, hence lungworm reports for sheep and goats must be considered together.

*Dictyocaulus filaria*, *P. rufescens*, *C. ocreatus*, and *M. capillaris* were reported in small ruminants in decreasing order (Figure 1B). The variable prevalence rate (0.1 to 63.6%) of lungworm infection in the reviewed studies may be due to numerous factors, including epidemiological influences of temperature, climate, host age and health status, and differences in the biology of different parasite species. The study inevitably revealed many reports of co-infections of *D. filaria* and *P. rufescens* and/or *C. ocreatus*.

In a newly published study, two first-stage larvae of *Muellerius* sp. were reported from the rehydrated fecal pellet of “sheep or goat” collected in historically rich Chehrabad salt mine in Iran’s northwestern Zanjan province, where several salt men and their personal belongings have been discovered [24]. It is difficult to speciate the animal origin of ancient small ruminant fecal pellets [25] however, this finding shows that this small ruminant lungworm was present in Iran since the era of the Sasanian Empire (500 CE).

**Table 1:** Lungworms of sheep and goats in Iran according to geographical area, nematode species and diagnostic method.

Geographical Region	Study Area	Nematode Species	Test	# Tested	% Prevalence	Host	Year of Study	References
**Northern Provinces**		**Single infections**						
	Tehran	*Dictyocaulus filaria*	Abattoir meat inspection	192	9.9	Sheep/Goat	1960–1961	[26]
	Tehran	*Dictyocaulus filaria*	Abattoir meat inspection	33	63.6	Sheep/Goat	Not stated	[27]
	Golestan	*Dictyocaulus filaria*	Abattoir meat inspection	60	5.0	Sheep	Not stated	[28]
	Golestan	*Dictyocaulus filaria*	Abattoir meat inspection	60	3.3	Goat	Not stated	[28]
	Semnan	*Dictyocaulus filaria*	Abattoir meat inspection	380	24.2	Sheep/Goat	2015	[29]
	Gilan	*Dictyocaulus filaria*	Baermann	140	7.1	Sheep	2017–2018	[30]
	Tehran	*Protostrongylus rufescens*	Abattoir meat inspection	192	24.5	Sheep/Goat	1960–1961	[26]
	Tehran	*Protostrongylus rufescens*	Abattoir meat inspection	33	9.1	Sheep/Goat	Not stated	[27]
	Golestan	*Protostrongylus rufescens*	Abattoir meat inspection	60	1.7	Goat	Not stated	[28]
	Semnan	*Protostrongylus rufescens*	Abattoir meat inspection	380	10.6	Sheep/Goat	2015	[29]
	Gilan	*Protostrongylus rufescens*	Baermann	140	2.1	Sheep	2017–2018	[30]
	Tehran	*Cystocaulus* sp.	Abattoir meat inspection	192	19.8	Sheep/Goat	1960–1961	[26]
	Tehran	*Cystocaulus ocreatus*	Abattoir meat inspection	33	3.0	Sheep/Goat	Not stated	[27]
	Gilan	*Cystocaulus ocreatus*	Baermann	140	1.4	Sheep	2017–2018	[30]
	Semnan	*Muellerius capillaris*	Abattoir meat inspection	380	1.5	Sheep/Goat	2015	[29]
	Gilan	*Muellerius capillaris*	Baermann	140	0.7	Sheep	2017–2018	[30]
		**Mixed infections**						
	Tehran	*Dictyocaulus* sp. *+ Protostrongylus* sp.	Abattoir meat inspection	192	2.6	Sheep/Goat	1960–1961	[26]
	Tehran	*D. filaria* + *P. rufescens*	Abattoir meat inspection	177	31.6	Sheep	1985–1986	[31]
	Tehran	*D. filaria* + *P. rufescens*	Abattoir meat inspection	33	9.1	Sheep/Goat	Not stated	[27]
	Tehran	*D. filaria* + *C. ocreatus*	Abattoir meat inspection	33	6.1	Sheep/Goat	Not stated	[27]
	Tehran	*C. ocreatus* + *P. rufescens*	Abattoir meat inspection	33	9.1	Sheep/Goat	Not stated	[27]
	Tehran	Lungworms larvae	Abattoir meat inspection	1627	3.7	Sheep	1997	[32]
	Golestan	Verminous pneumonia	Abattoir meat inspection	60	7.5	Sheep	Not stated	[28]
	Golestan	Verminous pneumonia	Abattoir meat inspection	60	7.5	Goat	Not stated	[28]
	Babol	Verminous pneumonia	Abattoir meat inspection	1000	1.6	Sheep	Not stated	[33]
	Semnan	*P. rufescens* + *M. capillaris*	Abattoir meat inspection	380	1.5	Sheep/Goat	2015	[29]
	Semnan	*D. filaria* + *P. rufescens*	Abattoir meat inspection	380	53.0	Sheep/Goat	2015	[29]
	Semnan	*D. filaria* + *M. capillaris*	Abattoir meat inspection	380	1.5	Sheep/Goat	2015	[29]
	Semnan	*D. f* + *P. r* + *M. c*	Abattoir meat inspection	380	7.6	Sheep/Goat	2015	[29]
**Western Provinces**		**Single infections**						
	Boroujerd	*Dictyocaulus filaria*	Abattoir meat inspection	175	12.6	Sheep	2008–2009	[34]
	Boroujerd	*Dictyocaulus filaria*	Abattoir meat inspection	140	9.3	Goat	2008–2009	[34]
	Hamedan	*Dictyocaulus filaria*	Abattoir meat inspection	100	9.0	Sheep	2010–2011	[35]
	Sanandaj	*Dictyocaulus filaria*	Abattoir meat inspection	164	8.5	Sheep	2017	[36]
	Sanandaj	*Dictyocaulus filaria*	Abattoir meat inspection	45	4.4	Goat	2017	[36]
	Boroujerd	*Protostrongylus rufescens*	Abattoir meat inspection	175	10.3	Sheep	2008–2009	[34]
	Boroujerd	*Protostrongylus rufescens*	Abattoir meat inspection	140	8.6	Goat	2008–2009	[34]
	Kermanshah	*Protostrongylus rufescens*	Abattoir meat inspection	492	0.4	Sheep	2013–2014	[37]
	Boroujerd	*Cystocaulus ocreatus*	Abattoir meat inspection	175	10.9	Sheep	2008–2009	[34]
	Boroujerd	*Cystocaulus ocreatus*	Abattoir meat inspection	140	7.9	Goat	2008–2009	[34]
	Kermanshah	*Muellerius capillaris*	Abattoir meat inspection	492	0.2	Sheep	2013–2014	[37]
		**Mixed infections**						
	Ilam	Lungworms larvae	Abattoir meat inspection	17,055	0.3	Sheep	2010–2011	[38]
	Kermanshah	Verminous pneumonia	Abattoir meat inspection	1200	3.4	Sheep	2013–2014	[39]
**Eastern Provinces**		**Single infections**						
	Mashhad	*Dictyocaulus filaria*	Abattoir meat inspection	2300	4.0	Sheep	2010–2011	[10]
	Mashhad	*Dictyocaulus filaria*	Baermann	320	7.2	Sheep	2010–2011	[10]
	Mashhad	*Dictyocaulus filaria*	Abattoir meat inspection	200	0.5	Goat	2010–2011	[10]
	Mashhad	*Dictyocaulus filaria*	Baermann	30	3.3	Goat	2010–2011	[10]
	Mashhad	*Protostrongylus rufescens*	Abattoir meat inspection	2300	0.3	Sheep	2010–2011	[10]
	Mashhad	*Protostrongylus rufescens*	Baermann	320	4.7	Sheep	2010–2011	[10]
**Northwestern Provinces**		**Single infections**						
	West Azarbaijan	*Dictyocaulus* sp.	Abattoir meat inspection	1325	1.5	Sheep	Not stated	[40]
	Urmia	*Dictyocaulus filaria*	Abattoir meat inspection	240	17.1	Sheep	1994–1995	[41]
	Urmia	*Dictyocaulus* sp.	Fecal flotation	1000	27.4	Sheep	1994–1995	[42]
	Urmia	*Dictyocaulus* sp.	Fecal flotation	580	24.8	Goat	1995–1996	[43]
	Urmia	*Dictyocaulus filaria*	Abattoir meat inspection	18,795	8.0	Sheep	Not stated	[44]
	Tabriz	*Dictyocaulus filaria*	Abattoir meat inspection	400	34.0	Sheep	2006–2007	[45]
	Tabriz	*Dictyocaulus filaria*	Baermann	1000	28.9	Sheep	2006–2007	[45]
	Shabestar	*Dictyocaulus filaria*	Abattoir meat inspection	712	0.7	Sheep	2008	[46]
	Tabriz	*Dictyocaulus filaria*	Abattoir meat inspection	235	26.8	Sheep	Not stated	[47]
	Salmas	*Dictyocaulus filaria*	Abattoir meat inspection	1922	0.1	Sheep	Not stated	[48]
	Zanjan	*Muellerius* sp.	Coprolite rehydration	1	Case report	Sheep or goat	Not stated	[24]
	Urmia	*Protostrongylus* sp.	Fecal flotation	1000	21.9	Sheep	1994–1995	[42]
	Urmia	*Protostrongylus* sp.	Fecal flotation	580	41.9	Goat	1995–1996	[43]
	Urmia	*Protostrongylus rufescens*	Abattoir meat inspection	18,795	8.4	Sheep	Not stated	[44]
	Tabriz	*Protostrongylus rufescens*	Abattoir meat inspection	400	11.0	Sheep	2006–2007	[45]
	Tabriz	*Protostrongylus rufescens*	Baermann	1000	12.7	Sheep	2006–2007	[45]
	Shabestar	*Protostrongylus rufescens*	Abattoir meat inspection	712	0.7	Sheep	2008	[46]
	Salmas	*Protostrongylus rufescens*	Abattoir meat inspection	1922	0.8	Sheep	Not stated	[48]
	Urmia	*Cystocaulus ocreatus*	Abattoir meat inspection	240	36.3	Sheep	1994–1995	[41]
	Urmia	*Cystocaulus* sp.	Fecal flotation	1000	32.9	Sheep	1994–1995	[42]
	Urmia	*Cystocaulus* sp.	Fecal flotation	580	38.3	Goat	1995–1996	[43]
	Urmia	*Cystocaulus ocreatus*	Abattoir meat inspection	18,795	8.6	Sheep	Not stated	[44]
	Tabriz	*Cystocaulus ocreatus*	Abattoir meat inspection	400	32.0	Sheep	2006–2007	[45]
	Tabriz	*Cystocaulus ocreatus*	Baermann	1000	29.4	Sheep	2006–2007	[45]
	West Azarbaijan	*Cystocaulus* sp.	Fecal flotation	403	0.8	Goat	2007	[49]
	Shabestar	*Cystocaulus ocreatus*	Abattoir meat inspection	712	3.9	Sheep	2008	[46]
	Meshkinshahr	*Cystocaulus ocreatus*	Abattoir meat inspection	90	2.2	Sheep	2010	[50]
	West Azarbaijan	*Muellerius* sp.	Abattoir meat inspection	1325	3.2	Sheep	Not stated	[40]
	Urmia	*Muellerius* sp.	Fecal flotation	1000	26.3	Sheep	1994–1995	[42]
	Urmia	*Muellerius* sp.	Fecal flotation	580	14.0	Goat	1995–1996	[43]
	Urmia	*Muellerius capillaris*	Abattoir meat inspection	18,795	26.0	Sheep	Not stated	[44]
	Tabriz	*Muellerius capillaris*	Baermann	1000	29.0	Sheep	2006–2007	[45]
		**Mixed infections**						
	Urmia	*D. filaria* + *P. rufescens*	Abattoir meat inspection	240	3.8	Sheep	1994–1995	[41]
	Urmia	*D. filaria* + *C. ocreatus*	Abattoir meat inspection	240	12.9	Sheep	1994–1995	[41]
	Urmia	*D. f* + *P. r* + *C. o*	Abattoir meat inspection	240	5.4	Sheep	1994–1995	[41]
	Urmia	Lungworms larvae	Abattoir meat inspection	2014	12.4	Sheep	2009	[51]
	Urmia	Verminous pneumonia	Abattoir meat inspection	626	3.7	Sheep	2011	[52]
**Southern Provinces**		**Single infections**						
	Shiraz	*Dictyocaulus* sp.	Baermann & abattoir meat inspection	102	21.1	Sheep	Not stated	[53]
	Fars	*Dictyocaulus* sp.	Abattoir meat inspection	579	22.6	Sheep	1991	[54]
	Shiraz	*Dictyocaulus filaria*	Abattoir meat inspection	1804	31.58	Sheep	2001–2005	[55]
	Kerman	*Dictyocaulus filaria*	Abattoir meat inspection	210	6.2	Sheep	2015–2016	[56]
	Shiraz	*Protostrongylus* sp.	Baermann & abattoir meat inspection	102	20.5	Sheep	Not stated	[53]
	Fars	*Protostrongylus* sp.	Abattoir meat inspection	579	19.3	Sheep	1991	[54]
	Shiraz	*Protostrongylus rufescens*	Abattoir meat inspection	1804	12.54	Sheep	2001–2005	[55]
	Kerman	*Protostrongylus rufescens*	Abattoir meat inspection	210	57.8	Sheep	2015–2016	[56]
	Fars	*Cystocaulus* sp.	Abattoir meat inspection	579	10.3	Sheep	1991	[54]
	Shiraz	*Cystocaulus ocreatus*	Abattoir meat inspection	1804	4.42	Sheep	2001–2005	[55]
	Shiraz	*Muellerius* sp.	Baermann & abattoir meat inspection	102	35.7	Sheep	Not stated	[53]
	Fars	*Muellerius* sp.	Abattoir meat inspection	579	26.8	Sheep	1991	[54]
	Shiraz	*Muellerius capillaris*	Abattoir meat inspection	1804	51.46	Sheep	2001–2005	[55]
**Southwestern Provinces**		**Single infections**						
	Ahvaz	*Dictyocaulus filaria*	Abattoir meat inspection	4592	0.2	Sheep	2008–2009	[57]
	Ahvaz	*Cystocaulus ocreatus*	Abattoir meat inspection	4592	0.3	Sheep	2008–2009	[57]
		**Mixed infections**						
	Ahvaz	*D. filaria* + *C. ocreatus*	Abattoir meat inspection	4592	0.1	Sheep	2008–2009	[57]
	Shahrekord	Verminous pneumonia	Abattoir meat inspection	1000	8.5	Sheep	Not stated	[58]
	Ahvaz	Verminous pneumonia	Abattoir meat inspection	4592	0.5	Sheep	2008–2009	[57]
**Samples from different regions**		**Single infections**						
		*Dictyocaulus filaria*	Abattoir meat inspection	30	13.3	Goat	Not stated	[59]
		*Dictyocaulus filaria*	Baermann	30	13.3	Goat	Not stated	[59]
		*Dictyocaulus filaria*	Abattoir meat inspection	135	24.4	Sheep	Not stated	[60]
		*Protostrongylus rufescens*	Baermann	30	20.0	Goat	Not stated	[59]
		*Protostrongylus rufescens*	Abattoir meat inspection	30	13.3	Goat	Not stated	[59]
		*Protostrongylus rufescens*	Abattoir meat inspection	135	22.2	Sheep	Not stated	[60]
		*Cystocaulus ocreatus*	Baermann	30	10.0	Goat	Not stated	[59]
		*Cystocaulus ocreatus*	Abattoir meat inspection	30	13.3	Goat	Not stated	[59]
		*Cystocaulus ocreatus*	Abattoir meat inspection	135	11.1	Sheep	Not stated	[60]
		*Muellerius* sp.	Baermann	30	3.3	Goat	Not stated	[59]
		*Muellerius capillaris*	Abattoir meat inspection	135	25.9	Sheep	Not stated	[60]
**Region not stated**		**Single infections**						
		*Dictyocaulus* *filaria*	Baermann	1712	41.0	Sheep	Not stated	[61]
		*Dictyocaulus* *filaria*	Abattoir meat inspection	99	56.0	Sheep	Not stated	[61]
		*Dictyocaulus* *filaria*	Baermann	297	21.0	Goat	Not stated	[61]
		*Dictyocaulus* *filaria*	Abattoir meat inspection	19	42.0	Goat	Not stated	[61]
		*Protostrongylus* sp.	Baermann	1712	26.0	Sheep	Not stated	[61]
		*Protostrongylus* sp.	Abattoir meat inspection	99	6.0	Sheep	Not stated	[61]
		*Protostrongylus* sp.	Baermann	297	16.0	Goat	Not stated	[61]
		*Protostrongylus* sp.	Abattoir meat inspection	19	16.0	Goat	Not stated	[61]
		*Muellerius* sp.	Baermann	1712	16.0	Sheep	Not stated	[61]
		*Muellerius* sp.	Abattoir meat inspection	99	9.0	Sheep	Not stated	[61]
		*Muellerius* sp.	Baermann	297	22.0	Goat	Not stated	[61]
		*Muellerius* sp.	Abattoir meat inspection	19	5.0	Goat	Not stated	[61]

### 3.2. Cattle and Buffalo Lungworms

Seven of 8 studies reporting lungworm infection in cattle and buffaloes were peer-reviewed journal articles, and one was a dissertation. These studies were conducted in the provinces of Mazandaran and Gilan in the north, East Azerbaijan and West Azerbaijan in the northwest, and Fars in the south. Overall, 1416 animals were assessed for the presence of lungworm infection, comprising 1127 cattle and 289 water buffaloes. For the diagnosis in cattle, 50.9% (574) of the cases were examined at abattoir meat inspection, 38.4% (433) were examined using the Baermann technique and 10.6% (120) were examined using fecal flotation. For the diagnosis in buffaloes, 34.6% (100) of the cases were examined at abattoir meat inspection and 65.4% (189) were examined using the Baermann technique. In total, 12.5% (54/433) and 5% (6/120) of tested cattle were found infected by the Baermann technique and fecal flotation, respectively and 17.5% (33/189) water buffaloes were found infected by the Baermann technique (Table 2). In 6 studies, the prevalence of *D. viviparus* was reported to range from 0.2 to 28.5% in cattle, and from 2.6 to 26.3% in buffalos. Moreover, in two reports, infection of cattle with *D. filaria* in the Fars and Mazandaran provinces was associated with co-grazing with sheep and goats [62,63] (Figure 1B).

### 3.3. Wild Ruminant Lungworms

Seven wild ruminant species are present in Iran, namely four species of the family Bovidae (goitered gazelle (*G. subgutturosa*), chinkara (*Gazella bennettii*), wild goat (*C. aegagrus*) and urial (*O. orientalis*)) and three species of the family Cervidae (Persian fallow deer (*Dama mesopotamica*), red deer (*C. elaphus*) and European roe deer (*C. capreolus*)). These species they are generally under protection in national parks or protected areas, and subject to close monitoring. The parasitic fauna of wild ruminants in Iran is poorly studied. Five peer-reviewed journal articles, one Master’s dissertation, and one conference paper contained information on lungworms of wild ruminants. Overall, 587 wild ruminant individuals belonging to five species were examined with an overall infection of 38.7% (227/587). 38% of urial, 37% of wild goats, 5% of goitered gazelles and 67% of red deer were reported to be infected with lungworms belonging to four genera of *Dictyocaulus*, *Protostrongylus*, *Cystocaulus* and *Muellerius*. A single case report described *Dictyocaulus* lungworm infection in a roe deer. 53.1% (312/587), 21.1% (124/587) and 17.2% (101/587) of cases were examined postmortem, by Baermann technique and fecal flotation, respectively. Urial have been studied more than the other wild ruminant species i.e., 367 urial were examined in four studies followed by 133 wild goats in three, 56 goitered gazelles in two, and 30 red deer in one studies. In one case report a single European roe deer was examined (Table 3). The reported prevalence of infection in urial ranged from 1% to 78% in different regions. Interestingly, high prevalences of *C. ocreatus* (94%), *D. filaria* (78%), and *Muellerius* sp. (70%) were reported in a study of Kaboodan Island on Urmia Lake, where animals *are* living in an isolated area without any external source of infection from domesticated animals [71].

### 3.4. Anthelminthic Drug Efficacy

Four studies, including 3 on sheep and goat lungworms and 1 on cattle lungworms have evaluated the efficacy of 9 anthelmintic active compounds (Table 4). Historically, methyridine (200 mg/kg PO), diethylcarbamazine (20 mg/kg IM), tetramisole (15 mg/kg PO), thiabendazole (100 mg/kg PO), and fenbendazole (20 mg/kg) were 100% effective for the treatment of *D. filaria* in sheep, while cyanacethydrazide (20 mg/kg SC) showed 83% efficacy [77]. The efficacies of thiabendazole (100 mg/kg PO), tetramisole (15 mg/kg PO), emetine hydrochloride (1 mg/kg PO) and fenbendazole (20 mg/kg) against *P. rufescens* were 97% (goats), 90% (goats), 91% (goats) and 100% (sheep), respectively [77]. The efficacies of thiabendazole (100 mg/kg PO), tetramisole (15 mg/kg PO), diethylcarbamazine (20 mg/kg IM) against *Muellerius* spp. in sheep were 56%, 26% and 28%, respectively; while the efficacies of thiabendazole (100 mg/kg PO), emetine hydrochloride (1 mg/kg PO) and emetine hydrochloride (3 mg/kg PO) against *Muellerius* spp. in goats were 46%, 68%, and 99%, respectively [77]. Fenbendazole (20 mg/kg PO) was 100% effective against *C. ocreatus* in sheep [78]. These anthelmintic drugs, with the exception of fenbendazole, are no longer licensed, or available for use in livestock. A single study evaluated a pour-on ivermectin formulation against *D. viviparus* in cattle, and reported efficacy of 99% [68].

## 4. Discussion

Around the world, lungworms are important causes of production inefficiency in pastoral livestock systems; albeit there has been a lack of critical evaluation of the impact of and rationale for control strategies in Iran. This study shows a high prevalence of lungworm in those provinces from which diagnostic information is available, consistent with the situation in neighboring countries, with similar climates, environments and pastoral animal management. For instance, in a study in Nangarhar, Afghanistan, 21.8% of 504 sheep examined in an abattoir were infected with lungworms [79], albeit the authors did not define the lungworm species. In Lahore, Pakistan, 31% of sheep and 11% of goats were found harboring lungworms, including *D. filaria*, *P. rufescens*, and *M. capillaris* [80]. *Dictyocaulus filaria* was also reported in 0.4% and 8.3% of sheep and goats, respectively, in the Chiltan National Park, Balochistan, Pakistan [81]. In Turkey, a relatively higher prevalence was reported in sheep, with 62.5% and 45.1% of animals testing positive in post-mortem and fecal examinations, respectively [79,82]. Regarding bovines, in neighboring Turkey, the prevalence of *D. viviparus* in cattle ranged between 0.3% and 70% [83], and in Faisalabad, Pakistan, the prevalence of *D. viviparus* infection in cattle and buffaloes was 4.8% and 5.1%, respectively [84]. *Dictyocaulus viviparus* lungworms present in Iran can have a major impact on cattle milk yields and growth rates, and cause death [5]. *Muellerius* and *Protostrongylus* spp. present in Iran also impact livestock production, in particular through the rejection of plucks at abattoir meat inspection [85]. This study, therefore, identifies a need to determine and implement appropriate, effective, and sustainable control strategies, based on a priori knowledge of the parasites’ life histories and contemporary understanding of the host, management and climatic factors influencing their epidemiology [86] under local Iranian conditions.

This study shows the presence of 7 species of lungworm in Iran, and the high prevalence of *D. viviparus* in cattle, and *D. filaria*, *Muellerius* and *Protostrongylus* spp. in sheep. The accuracy of species determination, in particular in mixed infections, must be interpreted with caution due to indistinct morphological traits of L_1_ [87]. The scarcity of published reports on cattle and buffaloes might not represent the true distribution and prevalence of lungworm infection in Iran. These hosts are widely distributed across the country, but due to ongoing droughts, the majority of cattle are housed with relatively good nutrition and limited exposure to infective lungworm L_3_; possibly accounting for the low prevalence of infection.

The control of lungworm infections in livestock is more challenging than that of gastrointestinal nematodes, not least because *Dictyocaulus* spp. infective L_3_ can be introduced by windborne spread, or survive for prolonged periods in the soil, while knowledge concerning the intermediate snail host specificity and distribution is lacking for *Protostrongylus* and *Muellerius* spp. The fundamental principal for control of *Dictyocaulus* spp. is to allow the establishment and maintenance of host immunity, which is rapidly acquired but requires lifelong boosting [88]. This usually requires the judicious use of anthelmintic drugs to reduce pasture contamination and thereby avoid high levels of infective L_3_ challenge. In some countries, this is complimented by vaccination [89]. However, these strategies are frequently unsuccessful in some regions, lungworms have become the predominant infectious disease causing production loss in cattle; putatively because climate change has extended periods of pasture growth and availability of infectious L_3_ [90]. In the absence of understanding of the factors influencing the epidemiology of *Muellerius* and *Protostrongylus* spp., control strategies are limited to the periodic use of anthelmintic drug treatments. Knowledge of drug efficacy against lungworm species is therefore, needed.

The modern-broad spectrum benzimidazole, imidazothiazole and macrocyclic lactone anthelmintic drugs are effective against L_4_ and adult stages of *Dictyocaulus* spp. in sheep and cattle [91]. In most countries, these drugs are not licensed for use in goats. However, there are no data sheet claims of efficacy of these drug groups against *Muellerius* and *Protostrongylus* lungworms. Currently, different anthelminthic products in forms of oral solution (levamisole, albendazole, fenbendazole, mebendazole, ivermectin, moxidectin), oral bolus (mebendazole) and injectable solution (ivermectin, doramectin) are labelled for treatment of lungworms in ruminants in Iran. This study included a single recent report of high efficacy of an ivermectin pour-on formulation against *D. viviparus* in cattle, and a historic report of high efficacy of an oral benzimidazole drug against *D. filaria* and *P. rufescens* in sheep. The no longer available, toxic and narrow spectrum anthelmintic drugs showed poor efficacy against *Muellerius* spp. (Table 4). There is, therefore, a need to demonstrate the efficacies of modern broad-spectrum anthelmintics against the lungworm species that are present in Iran. Drugs with efficacies less than 100%, can still be useful for lungworm control, but their use risks selection for anthelmintic resistance. Poor efficacy against certain parasite species may be due to inherent pharmacological properties of the drugs, which may be stage specific, for example differing between adults and migrating stages in the lymphatics [91].

Poor drug efficacy may indicate anthelmintic resistance; the emergence of which threatens the efficiency of food production from livestock irreversibly [92]. *Dictyocaulus viviparus* resistance to pour-on macrocyclic lactone drugs has been suggested, but not proven [93]. In Iran, there is growing evidence regarding resistance of trichostrongylid nematodes to common broad-spectrum benzimidazole and imidazothiazole compounds [94,95]. Many Iranian farmers do not adhere to recognized best practices of anthelminthic therapy [95], and resistance in gastrointestinal helminths has been reported [94,96]. There is, therefore, an urgent need to regularly evaluate different drug classes used in the treatment of lungworm infections, using studies designed according to international guidelines, and also respecting withdrawal periods for consumption and commercialization of milk and meat [97]. Strategies such as educating the farmers, combining anthelmintic drug classes, and developing antiparasitic vaccines and novel drugs could ameliorate the problem of anthelmintic resistance [95,98,99].

This study highlights the presence of lungworm infection in wildlife in Iran. It is important as wild species could act as reservoirs of infection for co-managed livestock [16]. While optimum strategies are needed for wild ruminant parasite control according to the diverse conditions of each region in Iran, the ethics and impacts of strategies such as non–intervention, or treatments with broad spectrum-drugs are unknown [100]. Furthermore, although it is known that different species of *Varestrongylus* and *Elaphostrongylus* parasitize the respiratory tracts of wild and domestic ungulates within the families Bovidae and Cervidae [101,102] these genera have not been recorded in wild ruminants of Iran. More focused studies with the aid of molecular techniques are first needed to explore lungworm infections in Iranian wildlife and co-managed pastoral livestock.

## 5. Conclusions

This work provides a framework for the development of effective and sustainable lungworm control strategies, and identifies areas of need for further research. Studies are needed to identify the environmental, climatic and management conditions that play a key role in the epidemiology of lungworm infection in Iran. These studies require the integration of conventional parasitology and molecular diagnostic methods for species level identification of lungworms, to inform cross-host species transmission, and reveal cryptic biodiversity. Large-scale studies should be designed to determine the prevalence of lungworm infection in Iran, to ensure the sustainability of control programs and mitigate against associated economic losses.

## Figures and Tables

**Table 2:** Lungworms of cattle and buffaloes in Iran according to geographical area, nematode species and diagnostic method.

Geographical Region	Study Area	Nematode Species	Test	# Tested	% Prevalence	Host	Year of Study	References
**Northern Provinces**		**Single infections**						
	Mazandaran	*Dictyocaulus filaria*	Abattoir meat inspection	1	Case report	Cattle	Not stated	[64]
	Gilan	*Dictyocaulus viviparus*	Baermann	212	22.6	Cattle	2018	[65]
	Gilan	*Dictyocaulus viviparus*	Abattoir meat inspection	212	2.4	Cattle	2018	[65]
	Gilan	*Dictyocaulus viviparus*	Baermann	189	26.3	Buffalo	2018	[65]
	Gilan	*Dictyocaulus viviparus*	Abattoir meat inspection	189	2.6	Buffalo	2018	[65]
	Mazandaran	*Dictyocaulus viviparus*	Abattoir meat inspection	1	Case report	Cattle	Not stated	[66]
**Northwestern Provinces**		**Single infections**						
	Urmia	*Dictyocaulus viviparus*	Abattoir meat inspection	470	0.2	Cattle	1996–1997	[67]
	Urmia	*Dictyocaulus viviparus*	Baermann	221	2.7	Cattle	1996–1997	[67]
	Urmia	-	Abattoir meat inspection	400	0	Buffalo	1996–1997	[67]
	Urmia	-	Baermann	135	0	Buffalo	1996–1997	[67]
	Tabriz	*Dictyocaulus viviparus*	Fecal flotation	120	5.0	Cattle	Not stated	[68]
	Tabriz	*Dictyocaulus viviparus*	Abattoir meat inspection	100	28.5	Cattle	2013	[69]
	Tabriz	*Dictyocaulus viviparus*	Abattoir meat inspection	100	19.0	Buffalo	2013	[69]
**Southern Provinces**		**Single infections**						
	Shiraz	*Dictyocaulus viviparus*	Abattoir meat inspection	1	Case report	Cattle	1998	[70]
	Shiraz	*Dictyocaulus filaria*	Abattoir meat inspection	1	Case report	Cattle	2003	[62]

**Table 3:** Reports on lungworm infections of wildlife ruminants.

Geographical Region	Study Area	Nematode Species	Test	# Tested	% Prevalence	Host	Year of Study	References
**Northern Provinces**		**Single infection**						
	Amol	*Dictyocaulus* sp.	Postmortem	1	Case report	*Capreolus capreolus*	2013	[63]
**Northwestern Provinces**		**Single infections**						
	Kabodan island-Urmia	*Dictyocaulus filaria*	Postmortem	50	78.0	*Ovis orientalis*	1994–1995	[72]
	Kabodan island-Urmia	*Protostrongylus rufescens*	Postmortem	50	38.0	*Ovis orientalis*	1994–1995	[72]
	Kabodan island-Urmia	*Cystocaulus ocreatus*	Postmortem	50	94.0	*Ovis orientalis*	1994–1995	[72]
	East Azarbaijan	*Muellerius capillaris*	Fecal flotation	30	40.0	*Capra aegagrus*	2015	[73]
	East Azerbaijan	*Dictyocaulus* sp.	Fecal flotation	30	66.6	*Cervus elaphus*	2015	[73]
	East Azarbaijan	*Cystocaulus* sp.	Baermann	74	36.0	*Capra aegagrus*	2016	[74]
		**Mixed infection**						
	Urmia	Lungworm larvae	Fecal flotation	41	34.1	*Ovis orientalis*	2002–2003	[71]
**National Park & protected regions of Iran**		**Single infections**						
	Not defined	*Dictyocaulus filaria*	Postmortem	250	14.4	*Ovis orientalis*	Not stated	[75]
	Not defined	*Dictyocaulus eckerti*	Postmortem	250	1.6	*Ovis orientalis*	Not stated	[75]
	Not defined	*Protostrongylus rufescens*	Postmortem	250	17.6	*Ovis orientalis*	Not stated	[75]
	Not defined	*Protostrongylus rufescens*	Postmortem	29	3.5	*Capra aegagrus*	2011–2012	[76]
	Not defined	*Protostrongylus rufescens*	Postmortem	32	3.0	*Gazella subgutturosa*	Not stated	[75]
	Not defined	*Protostrongylus raillietti*	Postmortem	250	4.8	*Ovis orientalis*	Not stated	[75]
	Not defined	*Cystocaulus ocreatus*	Postmortem	32	6.3	*Gazella subgutturosa*	Not stated	[75]
	Not defined	*Muellerius* sp.	Postmortem	250	1.1	*Ovis orientalis*	Not stated	[75]

**Table 4:** Anthelminthic efficacy tests on lungworms of ruminants in Iran.

Drug	Helminth Species	Host	Dose and Route of Administration ^a^	Efficacy (%)	References
Cyanacethydrazide	*Dictyocaulus filaria*	Sheep	20 mg/kg SC ^b^	83	[61]
Methyridine	*Dictyocaulus filaria*	Sheep	200 mg/kg PO ^c^	100	[61]
Diethylcarbamazine	*Dictyocaulus filaria*	Sheep	20 mg/kg IM ^d^	100	[61]
Tetramisole	*Dictyocaulus filaria*	Sheep	15 mg/kg PO	100	[61]
Thiabendazole	*Dictyocaulus filaria*	Sheep	100 mg/kg PO	100	[77]
Thiabendazole	*Muellerius* sp.	Sheep	100 mg/kg PO	56	[77]
Tetramisole	*Dictyocaulus filaria*	Sheep	5 mg/kg PO	86	[77]
			10 mg/kg PO	100	
Tetramisole	*Muellerius* sp.	Sheep	15 mg/kg PO	26	[77]
Diethylcarbamazine	*Muellerius* sp.	Sheep	20 mg/kg IM	28	[77]
Thiabendazole	*Protostrongylus rufescens*	Goat	100 mg/kg PO	97	[77]
Thiabendazole	*Muellerius* sp.	Goat	100 mg/kg PO	46	[77]
Tetramisole	*Protostrongylus rufescens*	Goat	15 mg/kg PO	90	[77]
Emetine hydrochloride	*Protostrongylus rufescens*	Goat	1 mg/kg PO	91	[77]
Emetine hydrochloride	*Muellerius* sp.	Goat	1 mg/kg PO	68	[77]
Emetine hydrochloride	*Muellerius* sp.	Goat	3 mg/kg PO	99.45	[77]
Fenbendazole ^e^	*Dictyocaulus filaria*	Sheep	20 mg/kg	100	[78]
			40 mg/kg	100	
			60 mg/kg	100	
			80 mg/kg	100	
Fenbendazole ^e^	*Protostrongylus rufescens*	Sheep	20 mg/kg	100	[78]
			40 mg/kg	100	
			60 mg/kg	100	
			80 mg/kg	100	
Fenbendazole ^e^	*Cystocaulus ocreatus*	Sheep	20 mg/kg	100	[78]
			40 mg/kg	100	
			60 mg/kg	100	
			80 mg/kg	100	
Ivermectin pour-on	*Dictyocaulus viviparus*	Cattle	0.5 mg/kg	99.21	[68]

^a^ In publication, ^b^ Subcutaneous, ^c^ Oral, ^d^ Intramuscular, ^e^ currently available and labelled for lungworm treatment.

## Data Availability

Not applicable.
